# Lack of Cultural Competency in International Aid Responses: The Ebola Outbreak in Liberia

**DOI:** 10.3389/fpubh.2017.00005

**Published:** 2017-01-31

**Authors:** Hannah Grace Southall, Sarah E. DeYoung, Curt Andrew Harris

**Affiliations:** ^1^College of Public Health, Institute for Disaster Management, University of Georgia, Athens, GA, USA

**Keywords:** public health education, global health, emergency management, response, mitigation, Ebola, Liberia

## Abstract

A cornerstone of effective disaster management is that response should always begin and end at the local level (1). The response to the Ebola virus disease (EVD) outbreak in Liberia, West Africa, was a combination of independent efforts by many nations and organizations. Many of these independent efforts ignored or were not able to work with the local levels of emergency management in Liberia. This oversight occurred because of the Liberian’s mistrust of both their government and foreign aid groups, as well as the lack of cultural competency demonstrated by the aid groups. The health-care and emergency management infrastructure in Liberia appeared to be non-existent at the beginning of the EVD outbreak. However, there were resources available at the community level: the Liberians and their culture. Although these resources were rarely used, there were some instances in which communities were included in response efforts. It was in these instances that possible improvements to international disaster response protocol were found.

## Introduction

Effective disaster management consists of a continuous cycle of four activities: mitigation, preparedness, response, and recovery (2). Liberia’s limited government and health-care infrastructure prevented both mitigation of hazards and preparedness for incidents. As a result, the response to the Ebola virus disease (EVD) outbreak was reactive rather than a coordinated effort of well thought out and exercised plans, policies, and procedures. Despite the fact that the outbreak’s management relied solely on response, key disaster management theories were disregarded throughout the international aid groups’ response to the outbreak.

Traditionally, incident response begins and ends at the local level (1, 2). It is when the resources of the localities are depleted that a scaled-up response occurs. Assistance is then requested by the local leaders (e.g., mayor, governor, etc.), prompting regional, state, federal, or international partners to become involved in response to supplement the locals. The issue in Liberia was that international aid groups supplanted rather than supporting the local level (3).

The utilization of local emergency response is a fundamental tenant of emergency management theory (4, 5). Organizing response through local groups is taught in many countries, including the United States, New Zealand, Canada, the United Kingdom, and many others (6). During the 2014 EVD response in Liberia, this practice was not actively utilized by international aid groups. Many potential resources were lost because of this oversight. The response to the EVD outbreak could have been improved by adapting traditional methods of emergency response to fit into the culture being served. Through the inclusion of cultural competency, historical knowledge, and involvement of community leaders in emergency management, it may be possible to create a methodology of response that can be used in future international aid (7). The following paper will analyze the 2014 EVD response in Liberia and postulate how improvements could have been made *via* adherence to the hallmark theory of emergency management: response begins and ends locally.

## Background

The 2014 EVD outbreak differed from previous EVD outbreak in Africa for three main reasons: (1) the outbreak occurred in West Africa (index case was in Guinea), (2) the outbreak centered in an urban environment, and (3) the volume of people who contracted the virus was more than all other outbreaks combined (8). EVD spread rapidly throughout metropolitan Guinea, Sierra Leone, and Liberia (8). As of April 2016, West Africa had reported 28,616 cases of confirmed, probable, and suspected EVD infected persons with 11,310 resulting mortalities (9).

Liberia faced many challenges in their attempt to control EVD transmission. Their public health and medical infrastructure was decimated following two civil wars and political instability. In fact, of the 293 public health facilities in Liberia, only 51 survived the wars and only 30 physicians were left to serve over 3 million people (10, 11). A 2010 study of Liberia’s public health and medical infrastructure found that only 6 of 19 health facilities in 4 Liberian counties had cell phone or radio capability (12). This lack of infrastructure was unresolved in the months and years leading up to the EVD outbreak.

Since the end of the second civil war, Liberia has relied primarily on foreign aid and donations to finance their health budget and strengthen the county’s infrastructure (10, 13). Most of the funding granted to the Liberian government was used to build the Liberian military in an effort to enforce political stability and promote national security for Liberian citizens (13). Unfortunately, this practice was viewed with suspicion by the Liberian public given the amount of civil unrest following the civil war.

Mistrust of Sirleaf’s administration by Liberians grew following her reelection. Many Liberians began to see similarities between her and other West African dictators. A stagnant education system, non-existent health-care infrastructure, unemployment rates as high as 80%, and the assignment of Sirleaf’s family members to important government positions contributed to this mistrust (13). Post-conflict Liberia has understandably continued to mistrust their government, despite a decade of what were purportedly democratic elections (13). The continued growth of the Liberian military and its involvement in enforcing quarantine during the EVD outbreak has only perpetuated this mistrust (13).

## International Response Issues Identified

### International Aid Leadership

International assistance was delayed throughout West Africa by the lack of leadership and coordinated response. Since 2006, the World Health Organization (WHO) has been transitioning from active response and practical disease control to a more passive role of setting guidelines and recommendations. This shift in the WHO’s policy was expedited by the overreaction to the H1N1 pandemic in 2009 (14). Ultimately, the WHO’s step back from proactive aid and immediate decision making led to hesitation in declaring the EVD outbreak a Public Health Emergency of International Concern (PHEIC) (15). While the legal qualifications for declaring an event a PHEIC were met in March 2014, the WHO did not do so until August (14, 16). This 5-month delay, in conjunction with the absence of health infrastructure in the region, led to unnecessary morbidity and mortality by unnecessarily extending the time of uncontrolled EVD transmission.

The WHO traditionally would have coordinated the international response but was hesitant to take an active role in the EVD outbreak control. The response was not coordinated. No one organization or entity was able to implement the use of Ebola treatment centers (ETCs) effectively throughout the region (14). In response to the WHO’s delay, the UN attempted to establish a unified international response; however, they were not prepared to manage the response of a multinational outbreak (14, 17). The thrown-together Liberian health-care infrastructure created by multiple aid groups fell apart quickly.

### Health-care Resources

Furthermore, the rapid spread of EVD throughout urban areas of Liberia was perpetuated by a number of problems faced by both local and international responders. These problems compounded the outbreak by preventing effective isolation (the removal of infected individuals from a population) and quarantine (the removal of exposed individuals from a population) (2). The use of ETCs in Liberia and the rest of Western Africa was vital to the reduction of EVD transmission during the outbreak. At the most severe points of the outbreak in Liberia, all health-care facilities were overcrowded (14).

The trust of Liberian citizens was further challenged by the attempts of international aid groups to reduce EVD transmissions and their inability to treat ill patients. The establishment of health-care facilities in Liberia’s affected urban areas was necessary to both treat diseased patients and isolate infected ones. Many foreign countries, non-governmental organizations, and other aid groups independently built ETCs—an effort loosely coordinated through the Liberian Ministry of Health and Social Welfare (14).

The first group to construct ETCs was Medecins Sans Frontieres (MSF). MSF had over 10 years of experience treating rural and technologically removed populations for EVD and Ebola-like diseases. ETC plans and supply lists were rapidly available when the EVD outbreak became evident. MSF ETCs were utilized effectively throughout the outbreak (14). Red Cross Red Crescent was similarly experienced in treating populations with EVD and was able to set up ETCs in September, 2014, early enough to effectively isolate and treat patients (14).

Mobile laboratories from the United States and the United Kingdom were established early enough in the outbreak to assist in the diagnosis of patients during the height of the outbreak (14). These labs aided in confirming EVD infection in patients (14). Additionally, the United Kingdom built mobile tents similar to those used by MSF—by November, 2014, six UK tents were operational in Sierra Leone (14).

In contrast, there were some aid efforts that were not as successful. The United States sent 3,000 soldiers to Liberia to construct ETCs (14). A total of 17 treatment centers were built, generating a total of 1,700 beds (17). Unfortunately, these facilities were not fully utilized because there were no health-care workers available in Liberia to staff the centers and the centers were completed after the peak of EVD infection in Liberia (14). The American aid organization Samaritan’s Purse had to close their ETC in Liberia when two aid workers became infected with EVD.

Regardless of whether successfully built and managed, ETCs in Liberia were overcrowded (14). Because of the lack of bed availability, supplies, and caregivers at ETCs in Liberia, many centers were not able to provide supportive care to patients (14). The sheer scale of the resources (e.g., physical beds, staff, IV fluids) needed to treat over 10,000 patients in Liberia is difficult to predict, let alone supply (9, 18).

Patients and their families quickly realized that the infected persons would be isolated in the ETCs, but that they would not be provided supportive care. This compounded the trust issues Liberians had for the government and international aid groups. This mistrust developed into an active avoidance of aid groups and ETCs in many communities, which led to the hiding of ill family members, non-compliance with isolation and quarantine protocols, and animosity toward aid groups (19).

### Engagement of Locals

Likewise, lack of information given to the public by international and national aid groups prevented locals from knowing how to help themselves, aid the ill, and prevent transmission within their communities (14). Public health education campaigns implemented in Liberia during the outbreak included community education about transmission, signs, and symptoms of EVD, the importance of isolating the infected persons, and how early diagnosis and treatment benefits the ill ones (20, 21). However, according to Abramowitz et al. (16), most of these public health messages focused on making sure people knew that the EVD outbreak was occurring, and not on what people could do to protect themselves from infection in areas without a functioning health-care system. During the height of the outbreak, communities knew what EVD was and how it was spread but did not know how to continue working and living while the disease spread throughout the country (e.g., how to take care of family members, how to transport the ill to ETCs, and how to keep themselves safe when the stand-up EVD infrastructure failed) (16).

Since the Liberians were unable to depend on aid groups (either due to local mistrust or the aid groups’ lack of ability), West Africans learned from experience to protect themselves from infection through avoidance of strangers, isolation of the ill, and avoidance of contact with the dead (14). However, the establishment of this knowledge in these communities was severely delayed by limited information. The lack of education on self-sufficiency for communities extended the amount of time it took to control the spread of the virus.

## Recommendations

### Utilize Locals and Local Culture

In November 2014, Liberia’s Ministry of Health, the WHO, and the World Bank invited Liberians living in Liberia’s capital, Monrovia, to participate in focus groups (22). Through these discussions, the groups learned that locals did not support a planned incentive scheme (a reward of US$5 for every EVD case reported in Monrovia) but had input on solutions that would fit the needs of their communities within their cultural principles. For example, the provision of food to families in quarantine would encourage the public to comply with EVD transmission reduction policies. Additionally, ensuring communication between family members and patients in ETCs would make keeping track of loved ones more probable. Likewise, offering psychological support to those that have lost family members would help communities recover, and including EVD infection survivors in all levels of community recovery would reduce stigmatization of survivors throughout Liberia (8, 22).

Input from 386 Monrovian community leaders identified what a community-based approach to the EVD outbreak would look like if constructed when outside resources were delayed or unavailable (16). The study elucidated many points, as follows:

1. Input from community leaders discovered gaps in the state and international responses
2. Surveillance needed to begin within communities and by community members
3. Input from community leaders created plans that sustain existing social structures, culture, and conflict histories.

Understanding the culture of a community can be advantageous to containing the spread of EVD. For example, utilization of gendered roles within the communities at risk creates a more streamlined approach to community surveillance and treatment in the absence of a well-supported health infrastructure (16). Supporting men and women in their culturally allocated roles by educating women in the treatment of family members, quarantine procedures, and personal protection can reduce the strain put on Liberia’s limited number of health-care facilities (16). Likewise, assigning male community members leadership roles in Ebola Task Forces and Block teams can prevent the spread of EVD throughout communities. Furthermore, giving otherwise unemployed, bored, and scared young men surveillance and community supply management roles can help prevent social conflict by giving them a sense of purpose within the community. This involvement would also increase trust, program sustainability, and response capacity (23). In these ways, creating outbreak controls through response at a micro-social scale can be achieved.

### Create Community-Developed Response Plans

This community-developed response plan should be expanded upon by aid groups. In order to utilize communities’ desire to autonomously help themselves, aid groups need to work with communities in order to successfully reduce morbidity and mortality. Most communities within Liberia have extremely limited resources. Therefore, it is necessary to utilize the resources available, the people within them, and the culture they embody. Doing this will ensure that community members that remain in the area have some knowledge of infectious disease prevention and therefore increase Liberia’s infrastructure and potentially reduce the amount of national and international aid required during future outbreaks.

The idea that communicating with community leaders during response efforts is beneficial to the overall outcome is supported by the actions of the Liberian Ministry of Health in the Fuamah District’s village of Mahwah (24). In this case, ambulance shortages prevented the removal of infected patients from their homes to ETCs, making community isolation necessary to prevent the spread of EVD to the rest of the district. Input from local leaders enabled the Fuamah District Ebola Taskforce to safely and effectively quarantine the entire village (24). The case study concluded that both the overall national response and individual communities benefited from the integration of community leaders in the planning of community quarantine (24).

## Conclusion

Disaster and emergency management theory states that response should always begin and end at the local level. Throughout the international response to the EVD outbreak, response was handled from a level far above the community—a method that may have exacerbated the transmission of EVD rather than reduce it. The lack of medical and public health infrastructure in Liberia created a challenge for communities but not an impossible one to overcome. Working with community leaders to understand the culture of communities and ethnic groups, as well as utilizing the human resources available could create a flow of surveillance information, increase the number of treated patients, and reduce the transmission of infectious disease through education of the public in future infectious disease outbreak. Cultural competency is a field that both emergency management and public health need to expand upon—especially when key theories and policies pivot on the ability to utilize local resources. When we next need to respond to an outbreak—or any disaster—at an international level, it is recommended that response involves the communities being affected. Furthermore, their culture should be recognized as an asset to bridging any gap between aid groups and the people they serve. The case of Ebola in Liberia demonstrates the urgent need for an intervention approach to future events that are specifically designed based on the cultural and social norms within the affected community.

## Author Contributions

HS wrote the original draft of the article. CH and SD revised and corrected the original draft.

## Conflict of Interest Statement

The authors declare that the research was conducted in the absence of any commercial or financial relationships that could be construed as a potential conflict of interest.

## References

[1] Robert T Stafford Disaster Relief and Emergency Assistance Act, P.L. 93-288 as Amended. Washington, DC: Federal Emergency Management Agency (2003).

[2] Coule PL Mitas JA, editors. Core Disaster Life Support. 3rd ed. American Medical Association (2010).

[3] Haddow G Bullock J Coppola DP Introduction to Emergency Management. Amsterdam; Boston: Elsevier/Butterworth-Heinemann (2013).

[4] Murphy BL Locating social capital in resilient community-level emergency management. Nat Hazards (2007) 41(2):297–315. 10.1007/s11069-006-9037-6

[5] Waugh WL Streib G Collaboration and leadership for effective emergency management. Public Adm Rev (2006) 66:131–40. 10.1111/j.1540-6210.2006.00673.x

[6] McEntire DA Mathis S Comparative politics and disasters: assessing substantive and methodological contributions. In: McEntire DA, editor. Disciplines, Disasters and Emergency Management: The Convergence and Divergence of Concepts, Issues and Trends from the Research Literature. Springfield, IL: Charles C Thomas Publisher (2007). p. 178–95.

[7] Andrulis DP Siddiqui NJ Gantner JL. Preparing racially and ethnically diverse communities for public health emergencies. Health Aff (2007) 26(5):1269–79. 10.1377/hlthaff.26.5.1269 17848436

[8] Alexander KA Sanderson CE Marathe M Lewis BL Rivers CM Shaman J What factors might have led to the emergence of Ebola in West Africa? PLoS Negl Trop Dis (2015) 9(6):e0003652. 10.1371/journal.pntd.0003652 26042592 PMC4456362

[9] 2014 Ebola Outbreak in West Africa – Case Counts. Centers for Disease Control and Prevention (2016). Available from: https://www.cdc.gov/vhf/ebola/outbreaks/2014-west-africa/case-counts.html

[10] Kruk ME Rockers PC Williams EH Varpilah ST Macauley R Saydee G Availability of essential health services in post-conflict Liberia. Bull World Health Organ (2009) 88:527–34. 10.2471/BLT.09.071068 PMC2897988 20616972

[11] Joint Needs Assessment. National Transitional Government of Liberia. United Nations/World Bank (2004). Available from: http://apps.who.int/disasters/repo/12605.pdf

[12] Forrester JD Pillai SK Beer KD Neatherlin J Massaquoi M Nyenswah TG Assessment of Ebola virus disease, health care infrastructure, and preparedness – four counties, southeastern Liberia, August 2014. MMWR Morb Mortal Wkly Rep (2014) 63(40):891–3. 25299605 PMC4584611

[13] Moran MH Surviving Ebola: the epidemic and political legitimacy in Liberia. Curr Hist (2015) 114(772):177–82.

[14] Kekulé AS Learning from Ebola Virus: How to Prevent Future Epidemics. Ipswich: MDPI Publishing (2015). p. 3789–97. 10.3390/v7072797 PMC4517126 26184283

[15] Ebola Interim Assessment Panel. Report of the Ebola Interim Assessment Panel. (2015). Available from: http://www.who.int/csr/resources/publications/ebola/ebola-panel-report/en/

[16] Abramowitz SA McLean KE McKune SL Bardosh KL Fallah M Monger J Community-centered responses to Ebola in urban Liberia: the view from below. PLoS Negl Trop Dis (2015) 9(4):e0003706 10.1371/journal.pntd.0003706 25856072 PMC4391876

[17] Butler D Global Ebola response kicks into gear at last. Nature (2014) 513(7519):469 10.1038/513469a 25254453

[18] Wong KK Perdue CL Malia J Kenney JL Peng S Gwathney JK Supportive care of the first 2 Ebola virus disease patients at the Monrovia Medical Unit. Clin Infect Dis (2015) 61(7):e47–51. 10.1093/cid/civ420 26021993

[19] Blackley DJ Lindblade KA Kateh F Broyles LN Westercamp M Neatherlin JC Rapid intervention to reduce Ebola transmission in a remote village – Gbarpolu county, Liberia, 2014. MMWR Morb Mortal Wkly Rep (2015) 64(7):175–8. 25719678 PMC5779598

[20] Summers A Nyenswah TG Montgomery JM Neatherlin J Tappero JW Challenges in responding to the Ebola epidemic – four rural counties, Liberia, August-November 2014. MMWR Morb Mortal Wkly Rep (2014) 63(50):1202–4. 25522089 PMC5779531

[21] Kobayashi M Beer KD Bjork A Chatham-Stephens K Cherry CC Arzoaquoi S Community knowledge, attitudes, and practices regarding Ebola virus disease – five counties, Liberia, September-October, 2014. MMWR Morb Mortal Wkly Rep (2015) 64(26):714–8. 26158352 PMC4584843

[22] Kutalek R Wang S Fallah M Wesseh CS Gilbert J Correspondence: Ebola interventions: listen to communities. Lancet Glob Health (2015) 3(3):e131 10.1016/S2214-109X(15)70010-0 25618243

[23] Lin H Liu T Song T Lin L Xiao J Lin J Community involvement in dengue outbreak control: an integrated rigorous intervention strategy. PLoS Negl Trop Dis (2016) 10(8):e0004919. 10.1371/journal.pntd.0004919 27548481 PMC4993447

[24] Nyenswah T Blackley DJ Freeman T Lindblade KA Arzoaquoi SK Mott JA Community quarantine to interrupt Ebola virus transmission – Mahwah village, bong county, Liberia, August-October, 2014. MMWR Morb Mortal Wkly Rep (2015) 64(7):179–82. 25719679 PMC5779591

